# Gingival Recession: Review and Strategies in Treatment of Recession

**DOI:** 10.1155/2012/563421

**Published:** 2012-10-02

**Authors:** Koppolu Pradeep, Palaparthy Rajababu, Durvasula Satyanarayana, Vidya Sagar

**Affiliations:** ^1^Department of Periodontics, Sri Sai College of Dental Surgery, Vikarabad, Andhra Pradesh, India; ^2^Department of Periodontics, Kamineni Institute of Dental Sciences, Narketpally, Andhra Pradesh, India

## Abstract

One of the most common esthetic concerns associated with the periodontal tissues is gingival recession. Gingival recession is the exposure of root surfaces due to apical migration of the gingival tissue margins; gingival margin migrates apical to the cementoenamel junction. Although it rarely results in tooth loss, marginal tissue recession is associated with thermal and tactile sensitivity, esthetic complaints, and a tendency toward root caries. This paper reviews etiology, consequences, and the available surgical procedures for the coverage of exposed root surfaces, including three case reports.

## 1. Introduction

Gingival recession is a problem affecting almost all middle and older aged to some degree. Gingival recession is the apical migration of gingival margin to the cementoenamel junction (CEJ). The distance between the CEJ and gingival margin gives the level of recession. Gingival recession can be caused by periodontal disease, accumulations, inflammation, improper flossing, aggressive tooth brushing, incorrect occlusal relationships, and dominant roots. These can appear as localized or generalized gingival recession. Recession can occur with or without loss of attached tissue. Gingival recession may effect in accentuated sensitivity because of the exposed dentin, it can be assessed by an appearance of a long clinical tooth and varied proportion of the teeth when compared with adjacent teeth. 

## 2. Prevalence

According to the US National Survey, 88% of seniors (age 65 and over) and 50% of adults (18 to 64) present recession in one or more sites; progressive increase in frequency and extent of recession is observed with increase in age [1]. 

In the youngest age cohort (30 to 39 years), the prevalence of recession was 37.8% and the extent averaged 8.6% teeth. In contrast, the oldest cohort, aged 80 to 90 years, had a prevalence of 90.4% (more than twice as high) and the extent averaged 56.3% teeth (more than six times as large) [2].

Gingival recession is associated with the presence of supragingival and subgingival calculus and showed that the lingual surfaces of the lower anterior teeth were most frequently affected in 20–34 year age group in Tanzanian adult population [3].

## 3. Etiology

### 3.1. Calculus

Association between gingival recession with supragingival and subgingival calculus can be noted because of inadequate access to prophylactic dental care [3].

### 3.2. Tooth Brushing

Khocht et al. showed that use of hard tooth brush was associated with recession [4]. 

### 3.3. High Frenal Attachment

This may impede plaque removal by causing pull on the marginal gingival [5].

### 3.4. Position of the Tooth

Tooth which erupts close to mucogingival line may show localised gingival recession as there may be very little or no keratinized tissue [6]. 

### 3.5. Tooth Movement by Orthodontic Forces

The movement of tooth such as excessive proclination of incisors and expansion of the arch expansion are associated with greater risk of gingival recession [7]. 

### 3.6. Improperly Designed Partial Dentures

The partial dentures which have been maintained or designed which cause the gingival trauma and aid in the plaque retention have the tendency to cause gingival recession [8].

### 3.7. Smoking

The people who smoke have more gingival recession than nonsmokers.

The recession sites were found on the buccal surfaces of maxillary molars, premolars, and mandibular central incisors [9]. 

### 3.8. Restorations

Subgingival restoration margins increase the plaque accumulation, gingival inflammation, and alveolar bone loss [10]. 

### 3.9. Chemicals

Topical cocaine application causes gingival ulcerations and erosions [11]. 

## 4. Consequences

### 4.1. Aesthetics

The appearance of tooth becomes unattractive [12]. 

### 4.2. Gingival Bleeding and Plaque Retention

The recession may be a site clinically which offers plaque retention. 

### 4.3. Hypersensitivity

Recession will uncover the cervical dentine. Hypersensitivity is usually of a sharp and short duration often associated with cold stimulus. The mechanism of hypersensitivity that is accepted is the hydrodynamic theory of pain, which states that the movement of dental fluid in the dentinal tubules triggers sensory nerve fibers in the inner dentine and dentinopulpal junction [13]. 

### 4.4. Caries

There may be a risk of the development of root caries as root surfaces are exposed to oral environment and aid in the withholding of plaque. Patients on periodontal maintenance with an average of 64.7 exposed root surfaces per patient; the mean number of caries lesions which were detected were 4.3 in a prevalence study [14]. 

## 5. Treatment

### 5.1. Restorations, Crowns, and Veneers

Crowns may be placed to widen the clinical crown which may camouflage the exposed root surface

### 5.2. Construction of Gingival Mask

Patients who have several teeth with recession may have unaesthetic appearance because of black triangles. In these cases, where surgical procedure is not appropriate, silicone flexible gingival veneer or mask may be used. 

### 5.3. Root Conditioning

Application of tetracycline HCL or citric acid to root surface before placement of soft tissue graft.

### 5.4. Frenectomy

When the recession is caused by frenal pull in those cases, frenectomy is advised. If appropriate hygiene aids do not enable the patient to maintain the area plaque free, then frenectomy is advised to give ease to entrance to the site [15]. 

### 5.5. Surgical Root Coverage Techniques

- Free epithelialised gingival graft [16].
- Subepithelial connective tissue graft [17].
- Semilunar flap [18].
- Coronally advanced flap [19].
- Guided tissue regeneration [GTR] [20].

## 6. Case Report 1

**Figure 1:**
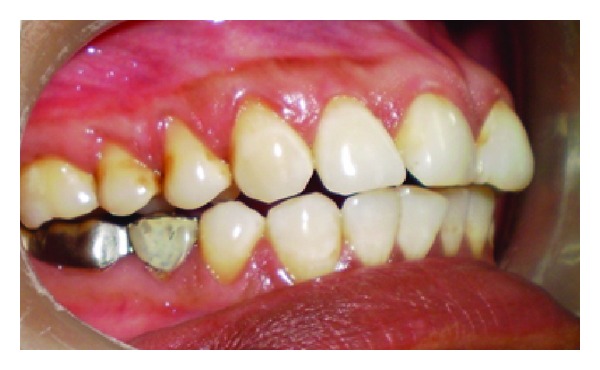
Facial view of gingival recession on 1st premolar and canine. The patient complained of root sensitivity in addition to the unaesthetic appearance when smiling.

**Figure 2:**
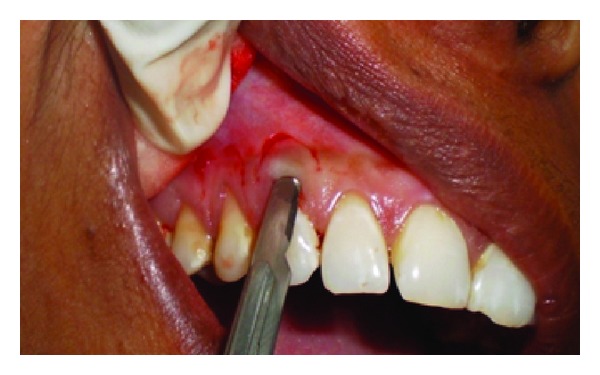
Semilunar incision placed apically.

**Figure 3:**
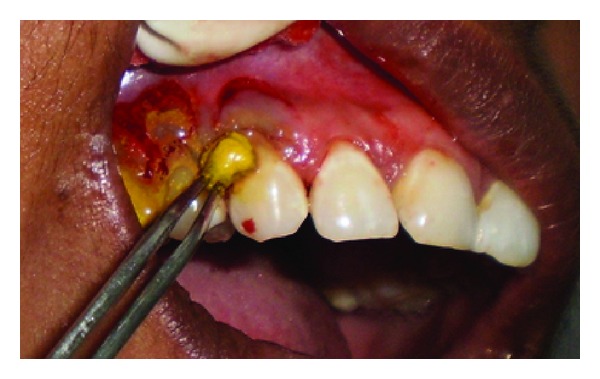
Root conditioning with tetracycline.

**Figure 5:**
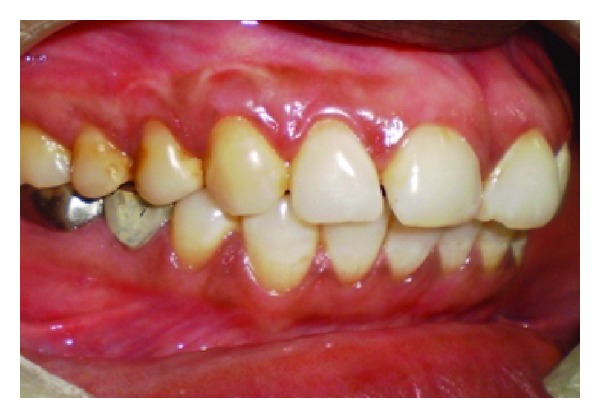
One -year postoperative view. Complete root coverage is observed.

A 43-year-old female patient complained of hypersensitivity inspite of using anti-hypersensitivity paste since 2 months and was also concerned about the esthetics. Patient had gingival recession on the maxillary left canine and first premolar at the first examination (Figure 1). The recession measured 2 mm on the canine and 3 mm on the first premolar, respectively. The clinical attachment loss was 4 mm from the CEJ for the canine and 5 mm for the first premolar, respectively. Oral prophylaxis has been done and oral hygiene instructions were given so as to achieve satisfactory plaque control prior to periodontal surgery. After reevaluation, a Semilunar incision and intracrevicular incisions have been given using Tarnow technique 22 (Figure 2). The roots were planed with hand curettes to remove the flecks of the calculus and to obtain smooth surfaces and then treated with tetracycline for 3 minutes (using a burnishing technique) (Figure 3). The root surfaces were then rinsed with saline. The flap was positioned as coronally as possible. The postoperative healing after 2 months revealed an increase of 2 mm and root coverage was achieved (Figure 5).

## 7. Case 2

**Figure 6:**
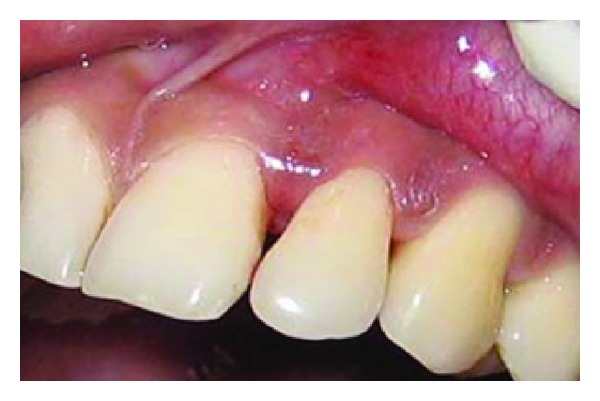
Blunting of interdental papilla between central and lateral incisor.

A 31-year-old female patient complained of a black triangle in the upper front teeth region since 6 months and was concerned about the esthetics and whistling sound while speaking (Figure 6). 

**Figure 7:**
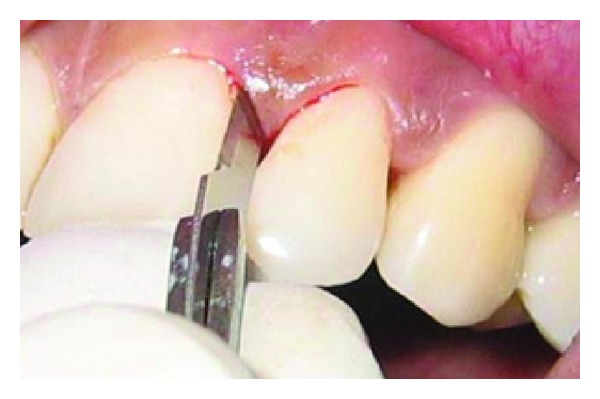
Intrasulcular incision.

**Figure 8:**
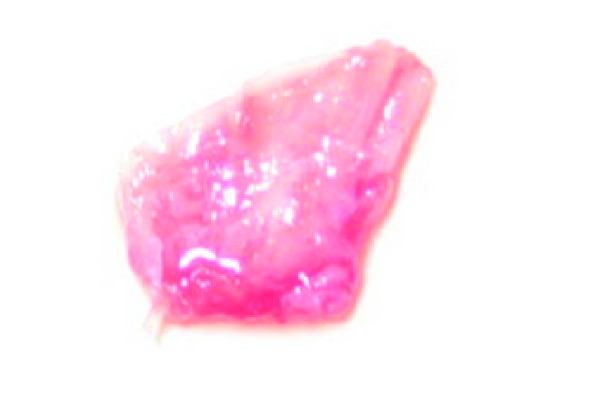
Connective tissue graft taken from palate.

**Figure 9:**
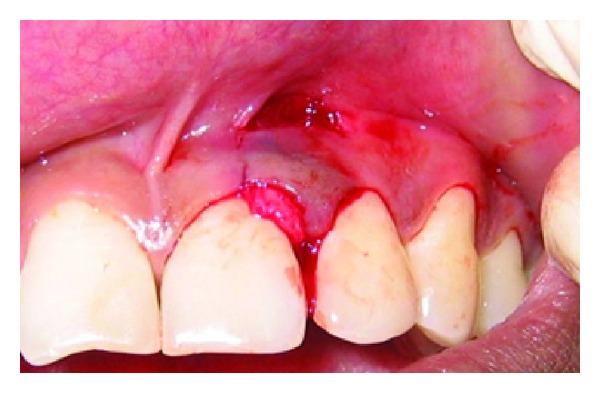
Graft placement under the flap.

**Figure 10:**
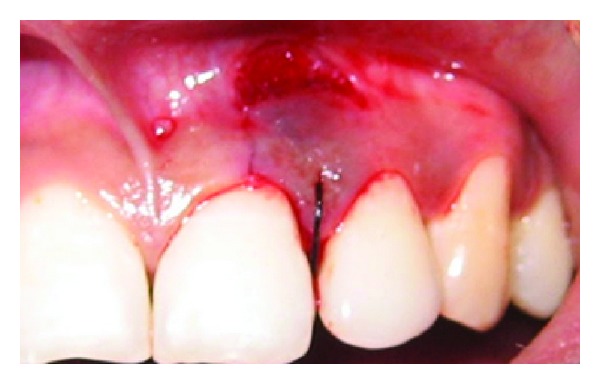
Placement of suture.

**Figure 11:**
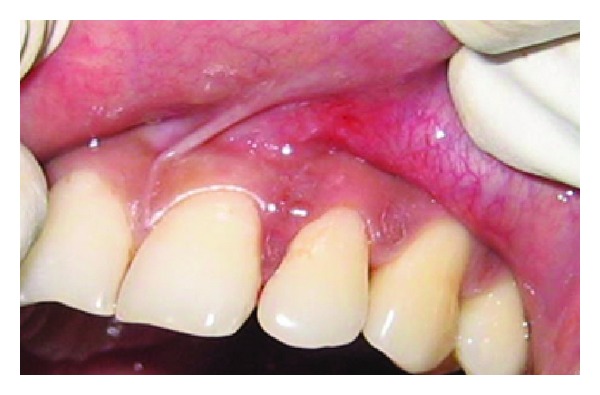
Closure of interdental papilla 6 months postoperatively.

The interdental papilla between maxillary right central and lateral incisor was blunt (Figure 6). Oral prophylaxis has been done and oral hygiene instructions were given so as to achieve satisfactory plaque control prior to surgery. An intrasulcular incision is made at the tooth surfaces facing the interdental area to be reconstructed (Figure 7), consequently an incision is placed across the facial aspect of the interdental area and an envelope-type, split-thickness flap is elevated; simultaneously, a semilunar incision was given apical to the mucogingival junction and the flap was coronally displaced using Langers technique. A connective tissue graft is harvested from palate (Figure 8) and placed under the flap in interdental area (Figure 9) and sutured back (Figure 10). In Figure 11 postoperative healing after 6 months revealed the excellent closure of black triangle between the left upper central and lateral incisors. 

## 8. Case 3

**Figure 12:**
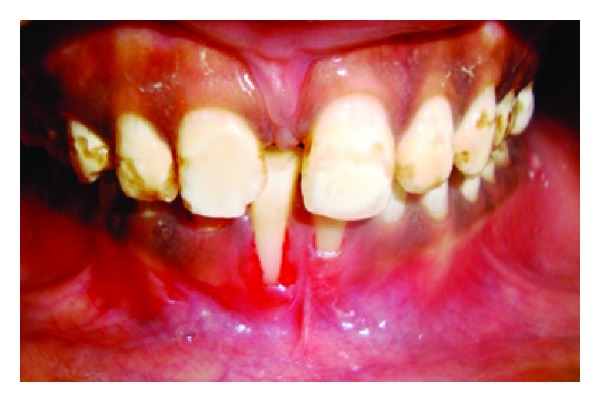
Recession of right central incisor.

**Figure 15:**
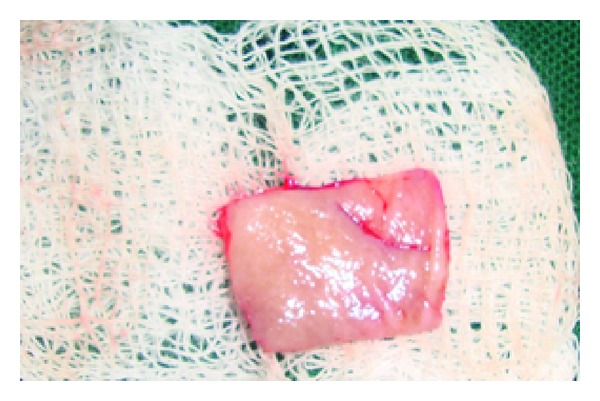
The donor tissue.

**Figure 16:**
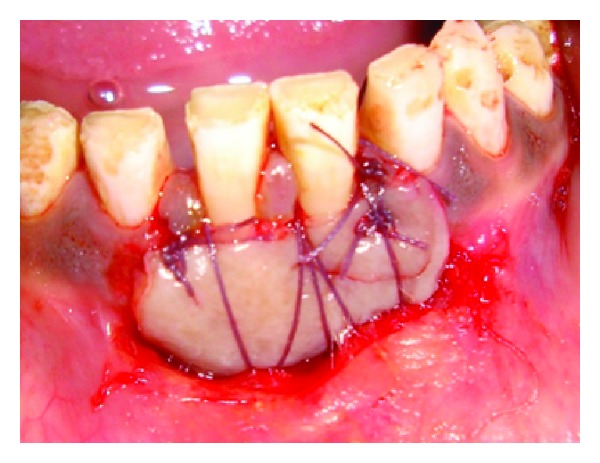
Suturing.

**Figure 17:**
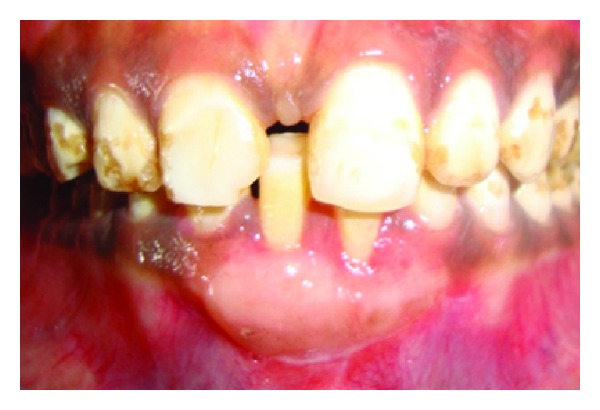
Six months postoperative healing.

A 25-year-old female patient complained of hypersensitivity in the lower front teeth region since 2 months and was concerned about the esthetics. Patient had gingival recession on the mandibular right central incisor at the first examination (Figure 12). The clinical attachment loss was 5 mm from the CEJ. Oral prophylaxis has been done and oral hygiene instructions were given so as to achieve satisfactory plaque control prior to periodontal surgery. The root surface was gently scaled and planed; instrumentation was done by utilizing manual and power driven scalers and curets. The shape of the root was not altered. The root surface was then treated with a tetracycline 500 mg by attempting to burnish, with small cotton pledgets. The donor tissue was removed from the palate and trimmed to a thickness of 2 to 3 mm (Figure 15). Within minutes of removal, the donor tissue was placed at the recipient site. Vertical stabilizing sutures (4–0 silk) were used to secure the graft (Figure 16). Postoperative healing after 6 months reveal excellent healing of the site and complete coverage of recession was achieved (Figure 17).

## 9. Discussion

The main goal of periodontal therapy is to improve periodontal health and thereby to maintain a patient's functional dentition right through his/her life. However, aesthetics symbolize an inseparable part of today's oral therapy, and numerous procedures have been proposed to preserve or enhance patient aesthetics. This treatment has principally been justified by the patient's wish to advance the aesthetic appearance when there is an exposed root.

Etiology and the contributing factors are chief when deciding on appropriate treatment procedures for patients with localized gingival recession. In the cases presented, the etiologies of the gingival recession were scarce vestibular depth and inadequate width of keratinized gingiva. If malposition of teeth is supposed to be the etiology for recession, then orthodontic treatment needs to be given a thought with or without periodontal therapy. Due to the existence of multiple mucogingival problems, it was decided to use a free gingival graft to accomplish root coverage and to form functional attached gingiva. The band of keratinized tissue was determined to be adequate in all cases. The color match and the tissue contour were satisfactory to the patients in all cases mentioned above. In some cases the color match and tissue contour match were good enough to make it complex to determine the position of the original defect.

The outcome of the current cases confirm aesthetics as the primary indication for root coverage. A recent survey showing that aesthetic concern was the foremost indication for root coverage procedures [21]. Indications other than aesthetic, root sensitivity were low and were grouped in the other category, accounting for 1.84% of the indications. 

## 10. Conclusion

Gingival recession is one of the main esthetic complaints of patients. This also exposes patients to sensitivity and greater risk for root caries. Mucogingival surgery endeavors' to reestablish the periodontium to a healthy circumstance. Periodontal plastic surgery strives to restore the periodontium to a healthy, efficient, and aesthetic state. For coverage of exposed roots, there is a vast range of mucogingival grafting procedures available in the present epoch. These procedures are quite predictable and produce satisfactory solutions to the problems presented by gingival recessions. Choice of appropriate procedure and surgical technique will recommend successful and exceedingly predictable results in the management of gingival recession.

**Key Finding(s)**

The present paper suggests that the selection of suitable procedure and specific and meticulous surgical technique will provide successful and exceedingly predictable results in the management of gingival recession.

## Figures and Tables

**Figure 4:**
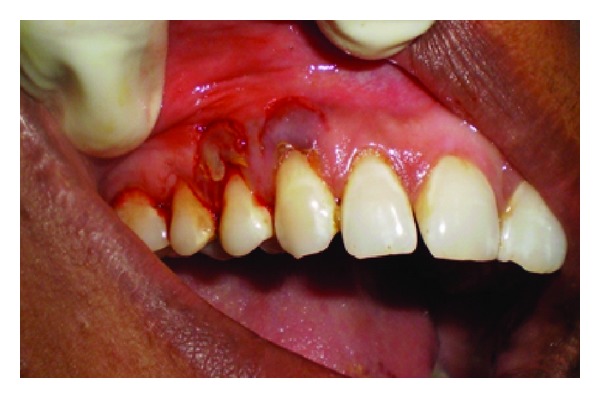
Repositioning of the flap coronally.

**Figure 13:**
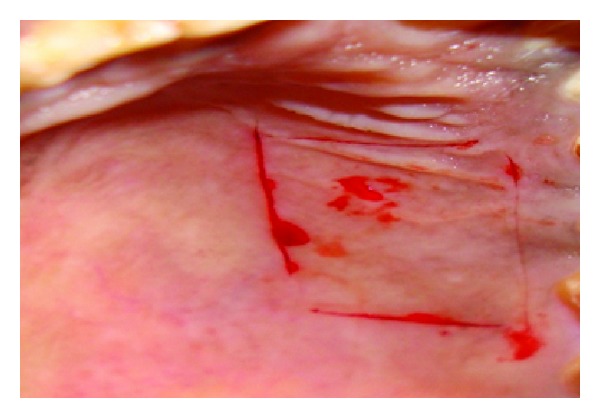
Incisions given for the removal of graft.

**Figure 14:**
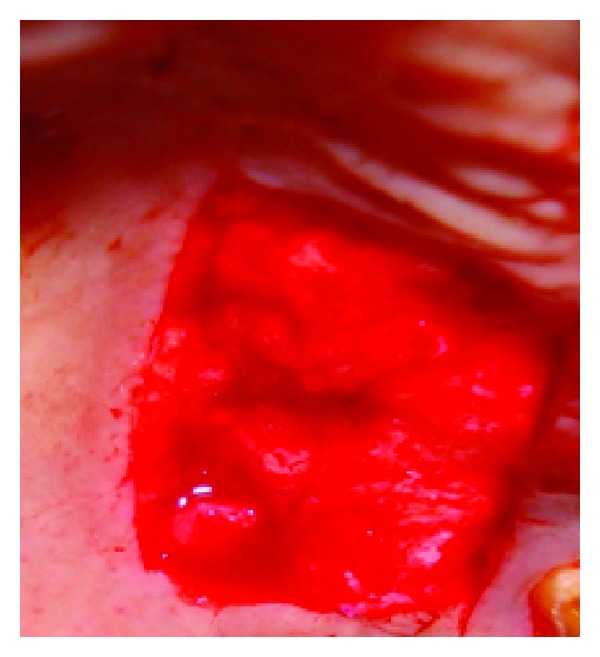
View of the palate after removal of the graft.
